# Longitudinal Changes in Pictorial Declarative Memory After Six Months of Clinical Management in Adults with Suspected Obstructive Sleep Apnea: A Prospective Cohort Study

**DOI:** 10.3390/clockssleep8030046

**Published:** 2026-08-12

**Authors:** Ainhoa Álvarez-Ruiz-Larrinaga, Jorge Ullate, Alejandro Horrillo-Maysonnial, Carla Pía, Carlos Egea-Santaolalla, David Gozal, Joaquin Durán-Cantolla, Maria Comas

**Affiliations:** 1 Bioaraba Health Research Institute, Neuroscience, Sleep Disorders, 01009 Vitoria-Gasteiz, Spain; jorge.ullatemora@bio-araba.eus (J.U.); alejandrojose.horrillomaysonnial@osakidetza.eus (A.H.-M.); piamartinezmd@gmail.com (C.P.); carlosegeasantaolalla@gmail.com (C.E.-S.); 2 Sleep Unit, Osakidetza Basque Health Service, Araba University Hospital, 01004 Vitoria-Gasteiz, Spain; 3 Department of Medicine, University of the Basque Country (UPV/EHU), 48940 Leioa, Spain; joaquin.durancantolla@gmail.com; 4 Centro de Investigación Biomédica en Red de Enfermedades Respiratorias (CIBERES), 28029 Madrid, Spain; 5 Departments of Pediatrics and Biomedical Sciences, Joan C. Edwards School of Medicine, Marshall University, Huntington, WV 25755, USA; gozal@marshall.edu; 6 Sleep Unit, Eduardo Anitua Medical Clinic, 01007 Vitoria-Gasteiz, Spain; 7 IKERBASQUE–Basque Foundation for Science, María Díaz de Haro 3, 48013 Bilbao, Spain

**Keywords:** declarative memory, sleep, obstructive sleep apnea, cognition, polysomnography

## Abstract

Background: Obstructive sleep apnea (OSA) has been implicated in declarative memory impairment. Here, we evaluated immediate recall and overnight declarative memory in patients with suspected OSA before and after standard clinical management in a longitudinal cohort of 265 patients. Methods: A nocturnal polysomnographic study (PSG) was performed at baseline and repeated six months later. The Pictorial-Based Declarative Memory Questionnaire (PDMQ) was administered and consisted of four learning trials followed by free-recall 10 min before PSG and the following morning upon awakening. The primary cognitive endpoint was the change in pictorial declarative memory performance from baseline to 6-month follow-up, expressed as the number of correctly recalled items on the PDMQ. Results: Both immediate and delayed recall performances significantly improved in the no OSA/mild OSA and severe OSA groups, whereas only immediate recall improved in the moderate OSA group. In the severe group, comparisons between patients whose apnea–hypopnea index decreased to <30 events/hour and those whose AHI remained >30 revealed significant cognitive improvements in both subgroups, particularly in initial and late trials (Trials 1, 2, 5, 6). In multivariable models, baseline PDMQ performance and indices of SpO_2_ desaturation and sleep fragmentation emerged as independent predictors of change in declarative memory. Conclusions: These findings suggest that routine clinical management of patients with OSA was accompanied by modest, albeit statistically significant improvements in declarative memory, and that both intermittent hypoxia and sleep fragmentation play important roles in memory change.

## 1. Introduction

Sleep plays an active role in memory consolidation by activating processes that stabilize and reorganize newly acquired information [1]. Memory is categorized into declarative and non-declarative [2]. During non-rapid eye movement (NREM) sleep, newly acquired knowledge is reactivated through slow-wave activity and sleep spindles, allowing for consolidation of declarative memories [3]. Rapid eye movement (REM) sleep is involved in memory processing and emotional memory. Beyond consolidation, sleep supports the integration of new memories into pre-existing neocortical schemas [4].

Obstructive sleep apnea (OSA) is a highly prevalent respiratory disorder that exerts a significant impact on overall health and increases the risk of cardiovascular [5], metabolic [6] and neurocognitive adverse consequences [7]. It is characterized by recurrent upper airway obstruction during sleep, leading to intermittent hypoxia, hypercapnia, disrupted sleep continuity and sympathetic activation. OSA is associated with alterations in sleep architecture, including disruption of REM sleep, a reduction in delta wave sleep (N3), along with decreased sleep spindle density [3].

Cognitive dysfunction associated with OSA is multifactorial [8,9,10,11,12,13]. Deficits in attention and vigilance are linked to increased arousals and accompanying sleep fragmentation, whereas impairments in cognitive function are primarily attributed to intermittent hypoxia [14]. Nevertheless, the precise mechanisms underlying memory dysfunction in OSA remain poorly understood [15].

In this prospective cohort study, we evaluated changes in pictorial declarative memory performance over a six-month period in adults referred for suspected OSA and examined whether these changes differed according to OSA severity. We hypothesized that current standard therapy [16] for OSA in adults would be associated with improvements in declarative memory function over time. We also explored whether baseline clinical and polysomnographic measures, including indices of sleep fragmentation and nocturnal hypoxemia, were associated with longitudinal changes in memory performance.

## 2. Results

### 2.1. Participant Flow and Baseline Characteristics

**Figure 1:**
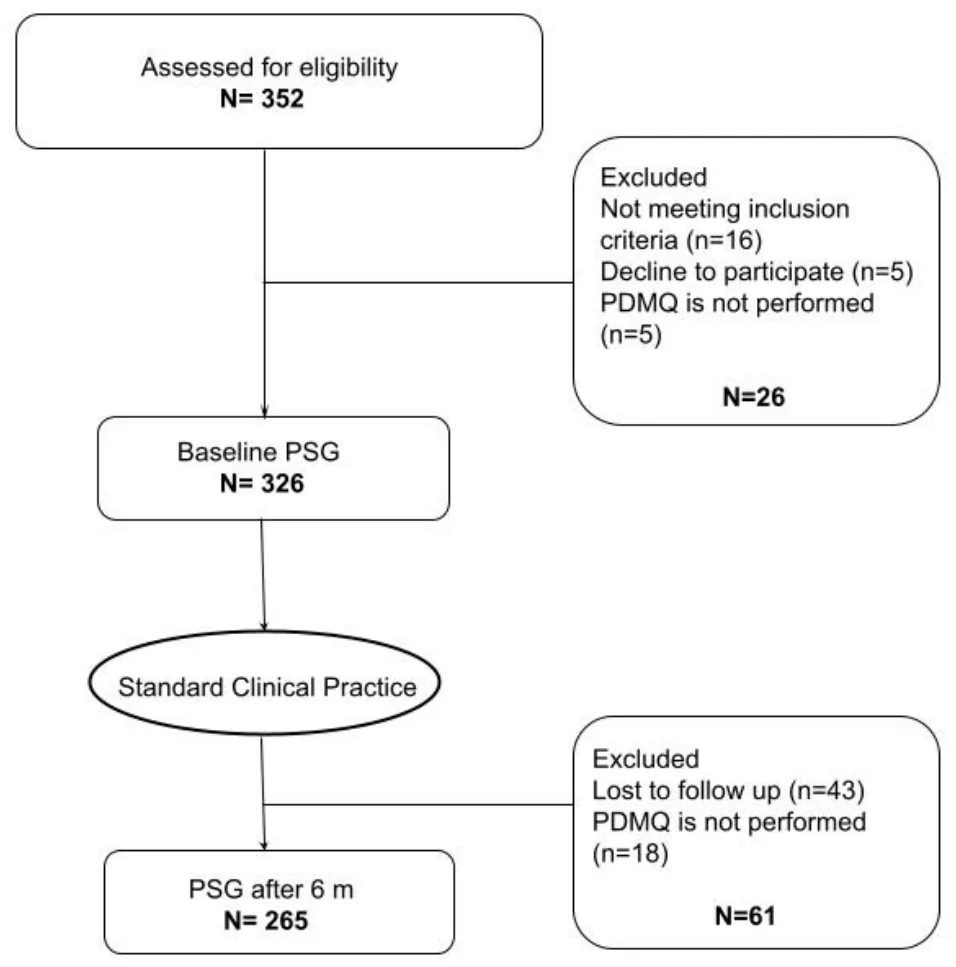
Study flow diagram. Of 352 patients assessed for eligibility, 26 were excluded (16 did not meet inclusion criteria, 5 declined to participate, 5 did not complete the Pictorial-Based Declarative Memory Questionnaire [PDMQ]). Baseline polysomnography (PSG) was performed in 326 patients. After standard clinical management and 6-month follow-up, 265 patients completed the second PSG and PDMQ; 61 were excluded (43 lost to follow-up, 18 did not complete the PDMQ).

Of the 352 patients initially evaluated for suspected OSA, 265 completed both baseline and 6-month evaluations and were included in the study (Figure 1). Based on baseline polysomnography, 80 participants were classified as no OSA/mild OSA (apnea–hypopnea index or AHI < 15 events/hour of sleep (e/h)), 59 as moderate (AHI 15–<30 e/h), and 126 as severe (AHI ≥ 30 e/h).

Table 1 summarizes the baseline demographic, anthropometric, and clinical characteristics of the participants according to OSA severity. The proportion of males increased with OSA severity (48.8% in no OSA/mild OSA vs. 72.9% and 78.6% in moderate and severe OSA, respectively; *p* < 0.001), as did mean age (48.6 ± 10.7 vs. 52.3 ± 10.0 vs. 55.6 ± 10.9 years, *p* < 0.001). Body mass index (BMI) and central adiposity also showed a clear gradient, with higher BMI, neck, and waist circumference in more severe OSA (all *p* < 0.001). Systolic and diastolic blood pressure were also higher in the severe group (both *p* < 0.001).

### 2.2. Clinical Management During Follow-Up

CPAP therapy was not indicated in the no/mild OSA group. In patients with moderate and severe OSA, CPAP was prescribed to 15.3% and 71.4%, respectively. The mean nightly CPAP use, objectively recorded by the device, was 6.5 ± 1.6 h, with no significant differences between groups (*p* = 0.686). Among the remaining patients with severe OSA, management followed routine clinical practice recommendations. Accordingly, 15.1% received conservative management with lifestyle recommendations (weight reduction, avoidance of alcohol and sedatives, and lateral sleeping position), whereas 3.2% were treated with positional therapy. CPAP was not prescribed when it was not clinically indicated or was declined by the patient. Six-month polysomnography (PSG) was performed under the prescribed treatment regimen when appropriate.

### 2.3. Neurocognitive Performance

**Figure 2:**
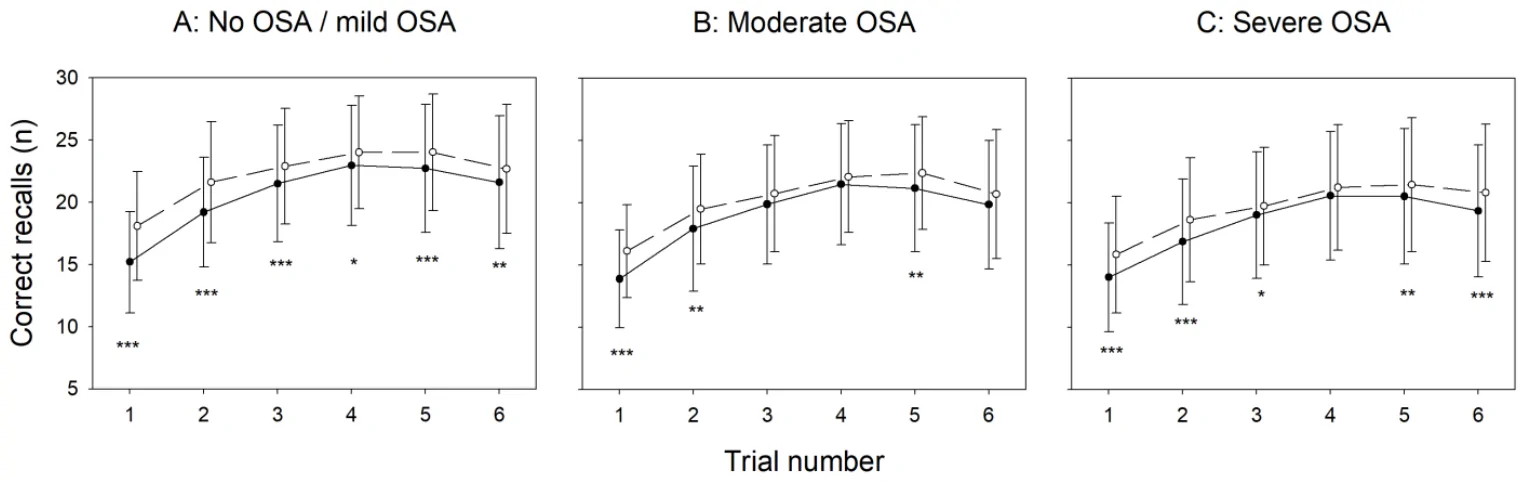
Pictorial-based declarative memory task performance by OSA severity. Mean number of correct recalls (±SD) at baseline (●, solid line) and at 6-month follow-up (○, dashed line) across trials 1–6 in (**A**) no OSA/mild OSA, (**B**) moderate OSA and (**C**) severe OSA. Asterisks under the symbols indicate significant within-group change between baseline and 6 months (* *p* < 0.05, ** *p* < 0.01, *** *p* < 0.001; paired *t*-test or Wilcoxon signed-rank test).

Performance on the PDMQ task is summarized in Table 2 and Figure 2. Across all OSA severity groups, memory performance showed modest but statistically significant improvements over the 6-month follow-up period. Within-group analyses demonstrated significant increases in the number of correctly recalled items in most trials, although the magnitude and consistency of these changes varied across groups. In the no OSA/mild OSA group, significant improvements were observed across all trials. In the moderate OSA group, significant pre–post differences were observed only in Trials 1, 2, and 5. In the severe OSA group, significant differences between baseline and 6-month scores were found in five out of six trials (Trials 1–3, 5, and 6).

Despite these within-group changes, no statistically significant differences in change scores (Δ) were observed between OSA severity groups, indicating that the magnitude of longitudinal improvement was broadly similar across groups.

However, cross-sectional comparisons at individual time points revealed a consistent gradient in performance. Participants with no OSA/mild OSA consistently exhibited higher mean recall scores than those with moderate or severe OSA, both at baseline and at 6 months. Several of these between-group differences reached statistical significance, particularly at 6 months.

Exploratory analyses were conducted in the severe OSA subgroup to examine whether longitudinal changes in memory performance differed according to treatment response, defined by improvement in apnea–hypopnea index (AHI) over the 6-month follow-up period (Supplementary Table S1). Participants were classified as improvers (AHI ≥ 30 to <30 events/h at follow-up) or non-improvers (AHI ≥ 30 at both time points). Within-group analyses revealed significant cognitive gains in both subgroups, particularly in the early trials. Improvers increased their number of correct recalls by an average of 1.51 ± 4.50 and 1.43 ± 4.23 in Trials 1 and 2, respectively (both *p* < 0.001). Additional but smaller improvements were observed in the immediate recall (Trial 5) and overnight delayed recall (Trial 6) (*p* = 0.018 and 0.004), whereas changes in Trials 3 and 4 did not reach statistical significance. Non-improvers also demonstrated significant improvement in the first two learning trials (Trials 1 and 2) (Δ = +2.87 ± 3.33 and +2.77 ± 3.78, both *p* < 0.001) and again in the overnight delayed recall trial (Trial 6) (*p* = 0.001). The intermediate trials showed no significant differences from baseline. When comparing change scores between subgroups (Δ = 6-month − baseline), no statistically significant between-group differences were detected in any of the six trials.

In multivariable analyses, baseline task performance was inversely associated with change in recall scores across all OSA severity groups and all six trials (all *p* < 0.001). Treatment status (treated vs. not treated) was not significantly associated with change in recall performance in any model after multivariable adjustment (all *p* > 0.05). Age showed inverse associations with change in several models, particularly in the moderate OSA group and, to a lesser extent, in severe OSA. No consistent associations were observed for sex (Supplementary Table S2).

### 2.4. Sleep Variables

Polysomnographic sleep macrostructure and sleep parameters at baseline and 6 months by OSA severity are summarized in Table 3. Total sleep time (TST) and sleep efficiency (SE) showed no statistically significant within-group differences in any severity group. Between-group comparisons of TST did not differ at either time point. SE was slightly lower in moderate and severe OSA at 6 months (*p* = 0.003), without relevant pre–post changes within groups. Wake after sleep onset (WASO) decreased significantly only in the no OSA/mild OSA group, although WASO was consistently higher in participants with more severe OSA at both assessments (*p* < 0.001).

Regarding sleep stage distribution, N1 sleep increased significantly from baseline in the no OSA/mild OSA and moderate groups (*p* = 0.023 and *p* = 0.019, respectively) but remained stable in the severe OSA group. N1 was consistently higher in the severe OSA group at both time points (*p* < 0.001 across severities). In contrast, N2 sleep decreased significantly from baseline to 6 months in all groups (*p* = 0.018, *p* < 0.001, and *p* < 0.001 for no OSA/mild OSA, moderate, and severe, respectively), with the largest decrease observed in participants with severe OSA (*p* = 0.019 across groups). Deep sleep (N3) showed a clear gradient across OSA severity. N3 increased significantly over time in the moderate and severe groups (*p* = 0.034 and *p* < 0.001) but showed only a non-significant trend in the no OSA/mild OSA group (*p* = 0.060). The overall proportion of non-REM sleep (N1 + N2 + N3) decreased significantly only in the severe group (*p* = 0.002), but no significant changes were observed in the other groups. Finally, REM sleep proportion increased significantly over time in the severe OSA group (*p* < 0.001) but remained stable in the mild and moderate groups. Both the total arousal index and the respiratory arousal index decreased significantly only in patients with severe OSA. The reduction in the respiratory arousal index in severe OSA was −13.21 (21.40) events per hour (*p* <0.001).

### 2.5. Polysomnographic and Physiological Correlates of Change in Declarative Memory Performance

Multivariable models examining polysomnographic and physiological variables are summarized in Supplementary Table S3. Across all OSA severity levels, baseline task performance remained a strong predictor of change across all six trials (all *p* < 0.001).

In the no OSA/mild OSA group, cognitive change was significantly associated with several sleep and oxygenation indices. A higher spontaneous arousal index predicted poorer improvement (β = −0.21, *p* = 0.019). Prolonged nocturnal oxyhemoglobin desaturation was the most consistent factor related to reduced gains, with both time spent with SpO_2_ < 85% and time spent with SpO_2_ < 90% showing strong negative coefficients (β ≈ −1.88 to −0.015, *p* < 0.03). Sex was also associated with change in Trials 2 and 3 (β = 1.816, *p* = 0.026; β = 2.335, *p* < 0.001).

In the moderate OSA group, lower nadir SpO_2_ and increased time with SpO_2_ < 90% were associated with reduced cognitive improvements, particularly in Trials 3 and 4 (*p* ≤ 0.002). Additionally, higher respiratory arousal index and BMI were both associated with smaller improvements, but only in Trial 5 (*p* = 0.007 and 0.002, respectively). Age also showed a significant negative association in some trials (e.g., Trials 2 and 5, *p* ≤ 0.03), indicating diminished recall gains in older participants.

In the severe OSA group, longer hypopnea duration (Trials 2 and 5; *p* ≤ 0.019) and obstructive apnea duration (Trial 4; *p* = 0.035) predicted smaller cognitive gains. In addition, longer REM sleep latency (Trial 5; *p* = 0.012) was inversely associated with improvement. Age was a significant predictor in Trials 1 and 4 (*p* = 0.015 and *p* = 0.034), with older participants showing smaller improvements. In contrast, treatment status, BMI, and sex were not significantly associated with cognitive change in any of the trials.

## 3. Discussion

This prospective cohort study investigated whether six months of OSA management were associated with changes in pictorial declarative memory performance, and whether these changes differed according to OSA severity. Memory performance improved modestly over follow-up in all severity groups, including both immediate recall and overnight delayed recall. However, the magnitude of these gains did not differ significantly between severities, and among patients with severe OSA, an exploratory subgroup analysis showed that those who improved their AHI over 6 months did not exhibit greater memory gains than those who remained with severe OSA. Overall, these findings suggest that changes in OSA severity alone are unlikely to be a major determinant of declarative memory improvements. In patients referred for suspected OSA, routine clinical management was accompanied by changes in sleep-related parameters and modest improvements in declarative memory performance. However, given the observational design and the absence of a sham-treated or independent non-OSA control group, these findings should be interpreted as longitudinal associations and not as evidence of a direct causal effect of treatment on memory.

Several explanations may account for the observed longitudinal improvement. Repeated administration of the same task may have contributed to practice effects, particularly during the evening learning trials. In our cohort, performance during the learning phase improved from baseline across all OSA severity groups, with significant differences in Trials 1 and 2 in all groups, in Trial 3 in the no OSA/mild OSA and severe OSA groups, and in Trial 4 in the no OSA/mild OSA group. This pattern could partly reflect a recall effect, since the PDMQ was administered for a second time after six months. However, memory tasks that are not revisited over extended periods of time tend to be rapidly forgotten, with retention typically ranging between 20% and 40% after several days [17]. In trials 2–4, significant differences in learning were observed both at the baseline visit and at the 6-month visit, which may reflect the effect of OSA-related hypoxemia on cognitive processing. Cross-sectional comparisons suggested lower overall memory performance in participants with more severe OSA. However, these analyses were unadjusted and must be interpreted cautiously, since the more severe groups were older and more frequently male.

Indices of nocturnal desaturation were associated with smaller gains in memory performance in several multivariable models. This pattern was most evident in the no OSA/mild OSA group, where longer baseline time with SpO_2_ < 85% and SpO_2_ < 90% was related to smaller gains in the number of correct responses during late learning trials and immediate recall (Trials 3–5, Supplementary Table S3). Similarly, although less consistent, associations between time with SpO_2_ < 90% and reduced improvement were observed in the moderate OSA group. This is in line with previous evidence linking executive function impairments with the severity of hypoxia and the occurrence of recurrent hypoxic events [18]. Büyükgök et al. [19] also reported impaired learning in a group of older adults with moderate to severe OSA (AHI > 15 e/h) compared with controls, highlighting the impact of OSA on the memory learning phase. In our cohort, the direction of the associations was generally consistent with a detrimental relationship between poorer nocturnal oxygenation and longitudinal change in task performance.

Memory consolidation has been proposed to depend on the structural organization of sleep and the extent of sleep fragmentation [20]. Our data show that sleep macrostructure changed after 6 months of routine clinical management, particularly in patients with moderate and severe OSA. NREM stage N3 sleep increased significantly in the moderate and severe OSA groups, with only a non-significant trend observed in the no OSA/mild OSA group, whereas REM sleep increased significantly only in the severe OSA group (Table 3). Recent studies provide further evidence for the crucial role of slow-wave sleep (SWS) in the consolidation of declarative memory [21]. Several mechanisms have been proposed to explain how N3 sleep contributes to memory consolidation, including the synaptic homeostasis hypothesis [22,23] or the low cholinergic tone characteristic of this stage [1]. However, in our multivariable models, the percentage of N3 did not emerge as an independent predictor of declarative memory temporal changes when adjusted by baseline performance, age, sex, BMI and treatment.

The disruption of sleep continuity observed in OSA may exert a stronger effect on memory performance than the relative distribution of sleep stages. We observed a significant reduction in both total arousals and respiratory arousal events in patients with severe OSA. However, the percentage of SWS did not emerge as an independent predictor of cognitive change in our multivariable models. Thus, in OSA, sleep fragmentation may exert a greater influence on memory than the relative proportion of sleep stages, a finding that has been extensively corroborated in animal models [11,24,25]. Overall, our findings emphasize the prominent role of nocturnal hypoxemia and sleep fragmentation in declarative memory performance in OSA.

### Strengths and Limitations

The main strengths of this study are the longitudinal design with PSG and cognitive testing at two time points, and the inclusion of patients covering the full spectrum of OSA severity under real-world sleep unit care. This approach allowed us to relate changes in declarative memory to both clinical management and detailed sleep and respiratory parameters.

Several limitations should also be acknowledged. The observational design and the absence of a sham-treated control group preclude causal inference and do not exclude placebo or practice effects. Analyses were conducted on complete cases. Therefore, loss to follow-up may have introduced selection bias, as participants who completed the study may not be fully representative of the initial cohort. Our findings should be interpreted in light of this potential source of bias. Treatment exposure and adherence were heterogeneous and followed routine clinical decision-making rather than a standardized protocol, limiting treatment-specific analyses, including those related to CPAP. Importantly, as this study was designed to evaluate cognitive changes following routine clinical management rather than the efficacy of any single treatment, it cannot determine which specific treatment modality, such as CPAP, lifestyle modification, or positional therapy, contributed most to the observed outcomes. Declarative memory was assessed with a single pictorial task, which may not generalize to other cognitive domains. Furthermore, repeated administration may have introduced practice effects, even though previous evidence suggests that unrehearsed material is largely forgotten over time [17]. Finally, residual confounding by unmeasured or insufficiently controlled factors, such as specific comorbidities, medication use, education level, or socioeconomic status, cannot be ruled out.

## 4. Materials and Methods

### 4.1. Study Design and Setting

This prospective longitudinal cohort study was conducted at the sleep unit of Araba University Hospital in Vitoria-Gasteiz, Basque Country, Spain, as part of the APNiA study [26]. Participants were recruited between September 2015 and March 2018.

### 4.2. Participants

Inclusion criteria were aged ≥ 18 years and referral for clinically suspected OSA. Exclusion criteria included previous evaluation of OSA, presence of another sleep disorder (insomnia, restless leg syndrome, circadian rhythm disorders, periodic limb movements in sleep), severe and unstable heart disease, or refusal to participate in the study. All patients provided written informed consent prior to their inclusion in the study. The study was conducted in accordance with the Declaration of Helsinki and received approval from the Clinical Research Ethics Committee (CEI) of Araba University Hospital in Vitoria-Gasteiz, Spain (approval code: BABTI-15-EC-01-APNIA; file: 2015-026; approval date: 18 September 2015).

### 4.3. Procedures

#### 4.3.1. Questionnaires

At baseline, demographic and anthropometric data were recorded, including age, sex and body mass index. Information on alcohol intake, smoking status, medication use and the presence of comorbid medical conditions was also recorded.

#### 4.3.2. Standard Clinical Treatment

Continuous positive airway pressure (CPAP) is the first-line treatment for moderate to severe OSA, as well as for mild OSA when clinically significant symptoms are present [16]. When CPAP is not indicated or is poorly tolerated, individualized alternative treatments may be considered, including mandibular advancement devices, positional therapy, and otolaryngology or maxillofacial surgery. All patients received guidance on lifestyle, dietary measures and management of potentially reversible comorbidities.

#### 4.3.3. Polysomnography

Overnight polysomnography (PSG) was performed at baseline and after six months, following standard recommendations in a sleep laboratory accredited by the Spanish Sleep Society, and staffed by certified somnologists as well as qualified nursing and technical personnel in sleep medicine [27,28]. Electroencephalogram (EEG) recordings included six EEG derivations (F3, F4, C3, C4, O1 and O2), referenced to contralateral mastoids (M1–M2) following the 10–20 rules of the international EEG system [27]. Electromyogram and electrooculogram were also recorded. Sleep stages (N1, N2, N3 and REM), arousals, oxygen saturation (SpO_2_) and apneas and hypopneas were scored using standardized guidelines [27,28]. OSA severity was classified using the apnea–hypopnea index (AHI) as no OSA (<5 events/h), mild OSA (5 to <15 events/h), moderate OSA (15 to <30 events/h), and severe OSA (≥30 events/h).

#### 4.3.4. Neurocognitive Tests

**Figure 3:**
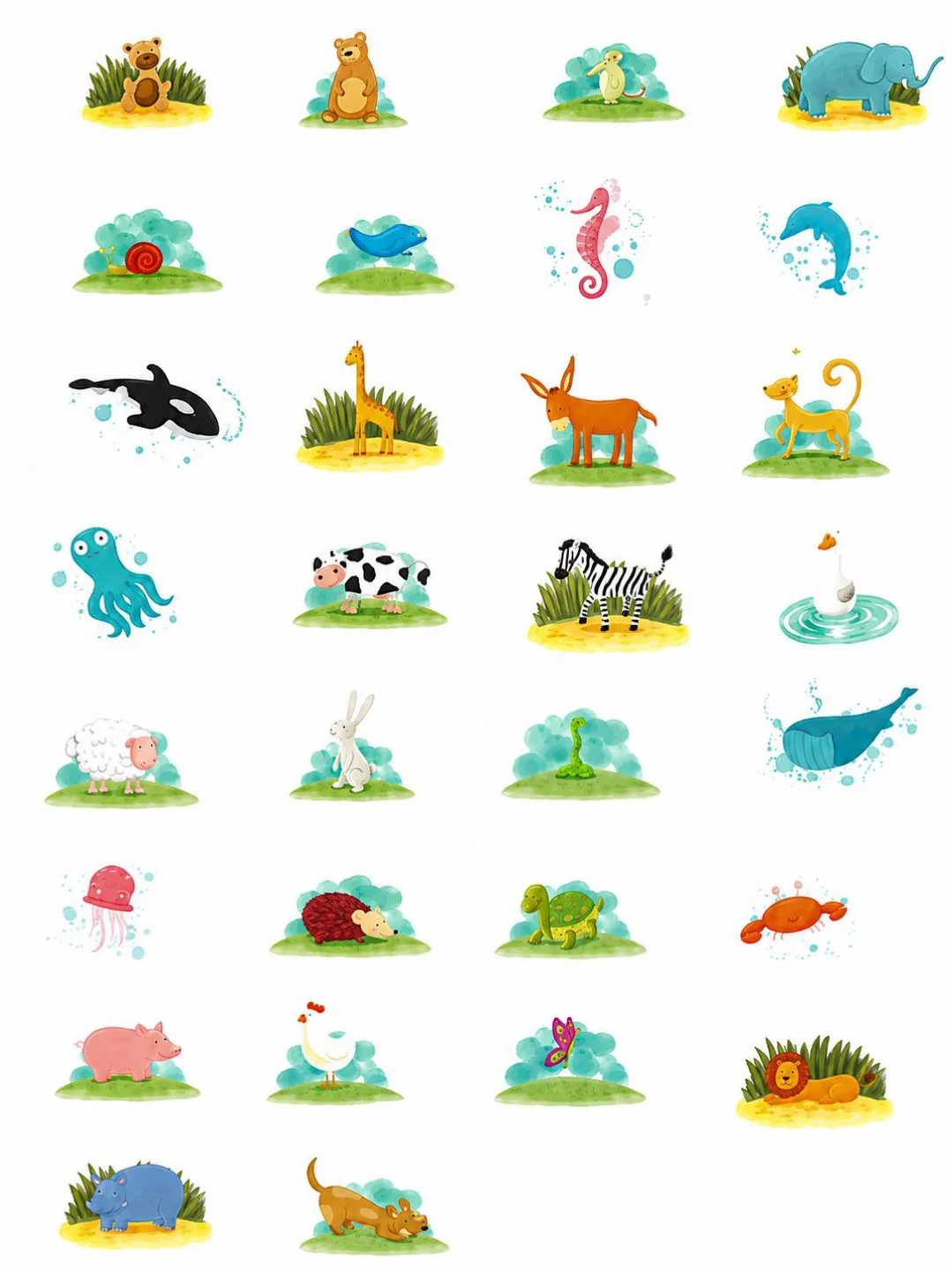
Animal pictures used in the Pictorial-Based Declarative Memory Questionnaire (PDMQ) [30]. The complete set of 30 animal pictures was presented during each of the four evening learning trials.

**Figure 4:**
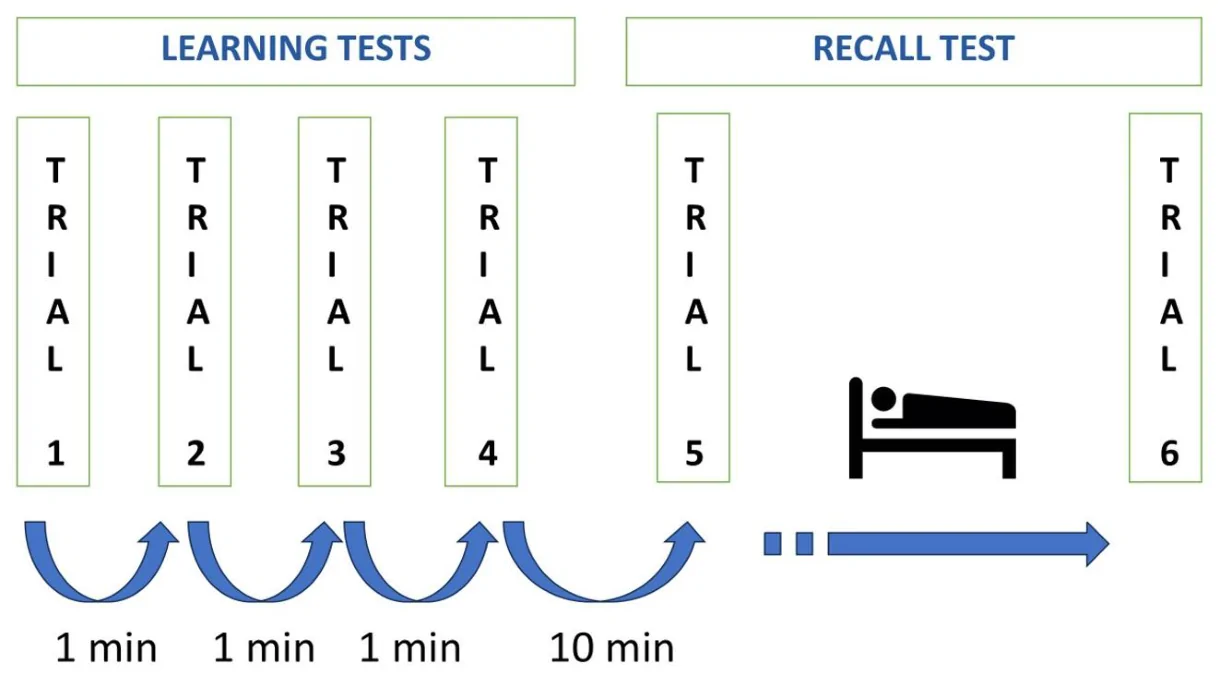
Schematic protocol of the pictorial-based declarative memory task. Participants completed four evening learning trials (Trials 1–4), each consisting of picture presentation followed by free recall and separated by a 1-min interval. An immediate recall trial was performed 10 min after the fourth learning trial (Trial 5), and a delayed recall trial was administered the following morning after overnight polysomnography (Trial 6).

Immediately before the overnight PSG, both at baseline and 6-month follow-up, declarative memory was assessed using the “Pictorial-Based Declarative Memory Questionnaire” (PDMQ) [29]. This declarative memory task originally designed for children was administered in adults without substantial modifications to preserve procedural comparability. Participants were shown a series of 30 animal pictures, each displayed for approximately 10 s (total presentation time 5 min) (Figure 3). They were then asked to freely recall as many animals as possible within 2 min. One point was awarded per correct response. This learning procedure was repeated four times (Trials 1–4), with a 1-min interval between consecutive trials. Ten minutes after completion of the fourth learning trial, participants performed an immediate free-recall test without being shown the pictures again (Trial 5). The following morning, within 10–15 min after awakening, they completed a second free-recall test (Trial 6), assessing overnight declarative memory consolidation (Figure 4). Trials 1–4 were considered learning trials, Trial 5 represented immediate recall, and Trial 6 represented overnight delayed recall.

### 4.4. Data Analysis

The primary cognitive endpoint was the longitudinal change in pictorial declarative memory performance between baseline and 6-month follow-up, assessed as the change in the number of correctly recalled items on the PDMQ. Trial-specific changes across the six PDMQ trials were analysed to characterize learning, immediate recall, and overnight delayed recall.

Numeric variables are presented as mean (SD), whereas categorical variables are presented as absolute frequencies (N) and percentages. Baseline characteristics were compared across OSA severity groups using parametric or non-parametric tests for continuous variables and chi-square or Fisher’s exact tests for categorical variables, as appropriate. Within-group changes from baseline to 6 months were assessed using paired *t*-tests or Wilcoxon signed-rank tests, as appropriate. Between-group differences in change scores were evaluated according to baseline OSA severity. The specific statistical test used in each comparison is indicated in the corresponding table footnotes. Analyses were restricted to participants with complete baseline and 6-month data. No imputation of missing data was performed. All statistical tests were two-sided with α = 0.05.

Exploratory multivariable linear regression analyses were performed to identify baseline clinical and polysomnographic factors associated with longitudinal changes in memory performance. Change in the corresponding PDMQ score was used as the dependent variable. Clinical models included age, sex, treatment status, and baseline PDMQ score. Separate exploratory models examining baseline physiological and polysomnographic variables, including indices of sleep fragmentation and nocturnal oxygen desaturation, were additionally adjusted for BMI.

No formal a priori sample size calculation was performed, as sample size was determined by the number of eligible patients recruited during the study period. All analyses were performed using R (version 4.5.1; 2025-06-13 ucrt) in a secure and validated cloud environment.

## 5. Conclusions

In this prospective cohort of adults referred for suspected OSA, routine guideline-based clinical management was associated with modest but consistent gains in pictorial declarative memory across all severity groups. Indices of nocturnal oxygen desaturation and sleep fragmentation, rather than changes in AHI or treatment type, were most strongly related to memory change, underscoring the importance of preserving nocturnal oxygenation and sleep continuity to support cognitive function in OSA. Further research is needed to compare treatment modalities, include broader neuropsychological assessments encompassing additional cognitive domains, and evaluate other factors that may influence outcomes, including detailed analysis of sleep microstructure (for example, sleep spindles) to provide deeper insights into the mechanisms underlying memory consolidation and cognitive function in patients with sleep disorders.

## Figures and Tables

**Table 1:** Baseline demographic, anthropometric and clinical characteristics by OSA severity groups. OSA severity was classified according to the Apnea–Hypopnea Index (AHI) obtained from overnight polysomnography (PSG). Continuous variables are reported as mean (SD) when approximately normally distributed, or as median [IQR] otherwise. Categorical variables are presented as *n* (%). The *p*-value column compares the three severity groups using the test indicated by the superscript: ^1^ Fisher’s exact test; ^2^ Chi-square test; ^3^ Kruskal–Wallis test; ^4^ ANOVA. All tests are two-sided (α = 0.05). Bold *p*-values indicate *p* < 0.05.

	Overall	AHI < 15	AHI 15–<30	AHI ≥ 30	*p*-Value
N	265	80	59	126	
Male sex, n (%)	181 (68.3)	39 (48.8)	43 (72.9)	99 (78.6)	**<0.001 ^1^**
Age (years)	52.5 (11.5)	48.6 (10.7)	51.2 (11.9)	55.6 (10.9)	**<0.001 ^4^**
BMI (kg/m^2^)	28.1 [25.7–31.1]	26.4 [24.3–29.1]	27.7 [25.8–30.1]	29.7 [26.7–32.2]	**<0.001 ^3^**
Neck circumference (cm)	39.8 (4.2)	37.45 (4.1)	39.16 (3.2)	41.6 (3.8)	**<0.001 ^4^**
Waist circumference (cm)	99.7 (12.8)	93.47 (13.0)	98.79 (11.0)	104.1 (11.8)	**<0.001 ^4^**
Systolic blood pressure (mmHg)	124.9 (16.0)	120.13 (16.0)	122.51 (13.4)	129.0 (16.2)	**<0.001 ^4^**
Diastolic blood pressure (mmHg)	78.0 (8.6)	74.7 (9.0)	77.58 (7.4)	80.2 (8.3)	**<0.001 ^4^**
Comorbidities, n (%)	163 (61.5%)	44 (55.0%)	36 (61.0%)	83 (65.9%)	0.305 ^1^
Cardiovascular	99 (63.1%)	25 (59.5%)	18 (56.3%)	56 (67.5%)	0.480 ^1^
Dermatological	4 (2.6%)	2 (4.8%)	1 (3.1%)	1 (1.2%)	0.413 ^1^
Gastrointestinal/Hepatic	25 (15.9%)	5 (11.9%)	3 (9.4%)	17 (20.5%)	0.292 ^1^
Nephro-urological	14 (8.9%)	2 (4.8%)	3 (9.4%)	9 (10.8%)	0.565 ^1^
Traumatological/Rheumatological	26 (16.6%)	4 (9.5%)	3 (9.4%)	19 (22.9%)	0.087 ^1^
Hematological	6 (3.8%)	1 (2.4%)	3 (9.4%)	2 (2.4%)	0.189 ^1^
Neurological	11 (7.0%)	5 (11.9%)	2 (6.3%)	4 (4.8%)	0.314 ^1^

**Table 2:** Pictorial-based memory task performance by OSA severity group and trial number (Trials 1–6). The table reports the mean (SD) number of correct recalls for each OSA severity group. For each trial (1–6), values are shown at three time points: Baseline (pre-PSG), 6-month follow-up, and Change (Δ), where Δ = 6-month − Baseline. Positive values in Change (Δ) indicate increases from baseline. Within each severity group, pre–post *p*-values reflect the paired comparison between baseline and 6-month. The final column (*p* across severities) tests whether Δ differs between severity groups. Statistical tests are indicated by superscripts: ^1^ ANOVA; ^2^ Wilcoxon signed-rank test; ^3^ Paired *t*-test; ^4^ Kruskal–Wallis test. All tests are two-sided (α = 0.05); bold *p*-values indicate *p* < 0.05. Paired analyses include only participants with available data at both time points.

Trial #	Time Point	AHI < 15	*p* Value Pre–Post (AHI < 15)	AHI 15–<30	*p* Value Pre–Post (AHI 15–<30)	AHI ≥ 30	*p* Value Pre–Post (AHI ≥ 30)	*p* Across OSA Severity Group
**1**	Baseline	15.18 (4.07)		13.86 (3.93)		13.99 (4.35)		0.091 ^1^
**1**	6-month	18.08 (4.37)		16.10 (3.74)		15.83 (4.68)		**<0.001 ^1^**
**1**	Change (Δ)	2.90 (3.83)	**<0.001 ^2^**	2.24 (3.53)	**<0.001 ^2^**	1.84 (4.27)	**<0.001 ^2^**	0.451 ^4^
**2**	Baseline	19.19 (4.41)		17.90 (5.04)		16.85 (5.04)		**0.003 ^1^**
**2**	6-month	21.60 (4.87)		19.47 (4.41)		18.61 (4.98)		**<0.001 ^1^**
**2**	Change (Δ)	2.41 (3.76)	**<0.001 ^3^**	1.58 (4.46)	**0.008 ^4^**	1.76 (4.15)	**<0.001 ^2^**	0.505 ^4^
**3**	Baseline	21.48 (4.69)		19.86 (4.78)		18.99 (5.07)		**0.002 ^1^**
**3**	6-month	22.88 (4.65)		20.71 (4.65)		19.71 (4.71)		**<0.001 ^1^**
**3**	Change (Δ)	1.40 (3.52)	**<0.001 ^3^**	0.85 (4.62)	0.164 ^3^	0.71 (3.72)	**0.033 ^3^**	0.455 ^1^
**4**	Baseline	22.95 (4.82)		21.47 (4.85)		20.55 (5.16)		**0.003 ^1^**
**4**	6-month	23.99 (4.52)		22.07 (4.48)		21.21 (5.03)		**<0.001 ^1^**
**4**	Change (Δ)	1.04 (3.94)	**0.021 ^3^**	0.59 (4.05)	0.265 ^3^	0.67 (3.87)	0.055 ^3^	0.7505 ^1^
**5**	Baseline	22.71 (5.14)		21.14 (5.10)		20.49 (5.44)		**0.013 ^1^**
**5**	6-month	24.01 (4.69)		22.36 (4.52)		21.44 (5.37)		**<0.001 ^1^**
**5**	Change (Δ)	1.30 (3.46)	**<0.001 ^3^**	1.22 (3.33)	**0.006 ^3^**	0.95 (3.78)	**0.005 ^3^**	0.770 ^1^
**6**	Baseline	21.59 (5.34)		19.83 (5.16)		19.33 (5.29)		**0.010 ^1^**
**6**	6-month	22.66 (5.17)		20.68 (5.19)		20.78 (5.52)		**0.028 ^1^**
**6**	Change (Δ)	0.97 (3.28)	**0.009 ^3^**	0.85 (3.48)	0.066 ^3^	1.45 (3.91)	**<0.001 ^3^**	0.485 ^1^

**Table 3:** Polysomnography-derived sleep macrostructure by OSA severity group at baseline and 6-month follow-up. For each sleep parameter, values at baseline, 6-month follow-up, and Change (Δ) are reported, where Δ = 6-month − Baseline. Positive values in Change (Δ) indicate increases from baseline. Values are presented as mean (SD) or median [IQR], as appropriate. Within each severity group, pre–post *p*-values reflect the paired comparison between baseline and 6-month. The final column (*p* across severities) tests whether Δ differs between severity groups. *p*-values are based on the following statistical tests: ^1^ ANOVA; ^2^ Kruskal–Wallis test; ^3^ Wilcoxon signed-rank test; ^4^ Paired *t*-test. All tests are two-sided (α = 0.05); bold *p*-values denote *p* < 0.05.

Parameter	Time Point	AHI < 15	*p* Pre–Post (AHI < 15)	AHI 15–<30	*p* Pre–Post (AHI 15–<30)	AHI ≥ 30	*p* Pre–Post (AHI ≥ 30)	*p* Across Severities
**Total Sleep Time (min)**	Baseline	357.62 (56.40)		342.78 (63.19)		352.48 (61.86)		0.358 ^1^
	6-month	363.49 (56.36)		360.24 (58.10)		359.14 (60.59)		0.872 ^1^
	Change (Δ)	5.86 (59.88)	0.383 ^3^	17.45 (69.07)	0.057 ^3^	6.66 (71.72)	0.299 ^3^	0.539 ^1^
**Sleep Efficiency (%)**	Baseline	81.84 (11.53)		79.39 (13.08)		78.61 (11.67)		0.163 ^1^
	6-month	83.12 (13.83)		81.37 (13.17)		78.66 (11.94)		**0.003 ^2^**
	Change (Δ)	1.27 (13.50)	0.401 ^3^	1.98 (13.30)	0.256 ^3^	0.05 (12.99)	0.968 ^3^	0.612 ^1^
**Sleep Onset Latency to NREM (min)**	Baseline	25.52 (21.73)		24.29 (22.19)		26.21 (22.09)		0.789 ^2^
	6-month	23.86 (21.24)		24.14 (31.42)		26.39 (24.33)		0.340 ^2^
	Change (Δ)	−1.66 (24.36)	0.543 ^3^	−0.15 (23.84)	0.937 ^3^	0.18 (26.44)	0.938 ^3^	0.875 ^1^
**WASO (min)**	Baseline	59.25 (45.79)		84.39 (142.56)		96.86 (256.79)		0.0974 ^2^
	6-month	47.91 (42.99)		61.72 (53.87)		71.74 (48.26)		**<0.001 ^2^**
	Change (Δ)	−11.34 (48.64)	**0.040 ^3^**	−21.77 (121.28)	0.159 ^4^	−25.13 (256.46)	0.924 ^4^	0.209 ^2^
**N1 Sleep (% of TST)**	Baseline	6.40 (3.76)		7.93 (3.86)		12.63 (10.47)		**<0.001 ^2^**
	6-month	7.58 (3.83)		10.63 (7.98)		13.80 (10.45)		**<0.001 ^2^**
	Change (Δ)	1.19 (4.59)	**0.023 ^3^**	2.70 (8.63)	**0.019 ^3^**	1.16 (12.92)	0.313 ^3^	0.594 ^1^
**N2 Sleep (% of TST)**	Baseline	58.03 (9.52)		58.85 (9.57)		60.52 (12.26)		0.258 ^1^
	6-month	54.38 (11.47)		52.33 (10.87)		51.21 (11.00)		0.139 ^1^
	Change (Δ)	−3.66 (13.63)	**0.018 ^3^**	−6.51 (13.13)	**<0.001 ^3^**	−9.30 (14.67)	**<0.001 ^3^**	**0.019 ^1^**
**N3 Sleep (% of TST)**	Baseline	17.26 (8.46)		16.53 (7.68)		11.64 (10.01)		**<0.001 ^2^**
	6-month	19.50 (9.19)		19.22 (7.86)		17.41 (8.95)		0.188 ^1^
	Change (Δ)	2.23 (10.50)	0.060 ^4^	2.69 (9.53)	**0.034 ^4^**	5.77 (10.92)	**<0.001 ^4^**	**0.035 ^1^**
**NON-REM Sleep (% of TST)**	Baseline	81.34 (5.76)		83.29 (5.85)		84.78 (6.13)		**<0.001 ^1^**
	6-month	81.46 (5.25)		82.33 (6.92)		82.44 (5.91)		0.493 ^1^
	Change (Δ)	0.12 (7.20)	0.883 ^3^	−0.96 (8.89)	0.409 ^3^	−2.26 (8.09)	**0.002 ^3^**	0.112 ^1^
**REM (% of TST)**	Baseline	18.31 (4.84)		16.71 (5.85)		15.24 (6.13)		**<0.001 ^1^**
	6-month	18.54 (5.25)		17.82 (6.51)		17.56 (5.90)		0.501 ^1^
	Change (Δ)	0.23 (6.53)	0.756 ^4^	1.11 (8.01)	0.291 ^4^	2.33 (8.20)	**<0.001 ^4^**	0.153 ^1^
**Total Arousal Index**	Baseline	9.85 [5.77–13.08]		17.80 [9.35–22.65]		25.60 [12.20–41.00]		**<0.001 ^2^**
	6-month	12.20 [9.17–15.90]		15.70 [12.20–23.85]		20.85 [11.43–32.15]		**<0.001 ^2^**
	Change (Δ)	2.40 [−1.80–7.20]	0.287 ^4^	0.50 [−6.80–8.20]	0.457 ^4^	−2.60 [−16.80–8.50]	**0.040 ^3^**	**0.030 ^2^**
**Respiratory Arousal Index**	Baseline	4.75 (3.01)		10.69 (5.02)		26.01 (20.03)		**<0.001 ^1^**
	6-month	6.45 [3.72–8.60]		9.20 [4.62–14.47]		9.00 [3.60–16.77]		0.058 ^2^
	Change (Δ)	2.60 (4.08)	**0.002 ^4^**	−1.59 (8.21)	0.332 ^4^	−13.21 (21.40)	**<0.001 ^4^**	**<0.001 ^1^**

## Data Availability

The data that support the findings of this study are available from the corresponding author upon reasonable request.

## Supplementary Materials

The following supporting information can be downloaded at: https://www.mdpi.com/article/10.3390/clockssleep8030046/s1, Table S1: Pictorial-based memory task (number of correct recalls) in the severe OSA subgroup: Improvers (AHI ≥ 30→<30 at 6 months) vs. Non improvers (AHI ≥ 30→≥30); Table S2: Associations between demographic and clinical variables and cognitive improvement over 6 months (Model I); Table S3: Associations between physiological sleep variables and memory task improvement over 6 months.

## Abbreviations

The following abbreviations are used in this manuscript:

AHI	Apnea–Hypopnea Index
BMI	Body Mass Index
EEG	Electroencephalogram
NREM	Non-Rapid Eye Movement
OSA	Obstructive Sleep Apnea
PDMQ	Pictorial-Based Declarative Memory Questionnaire
PSG	Polysomnography
REM	Rapid Eye Movement
SpO_2_	Peripheral Oxygen Saturation
SD	Standard Deviation
SWS	Slow-Wave Sleep
TST	Total Sleep Time
SE	Sleep Efficiency
WASO	Wake After Sleep Onset
