# Supplementary material for: Longitudinal Changes in Pictorial Declarative Memory After Six Months of Clinical Management in Adults with Suspected Obstructive Sleep Apnea: A Prospective Cohort Study
Source: Clocks Sleep. 2026 Aug 12;8(3):46. doi: 10.3390/clockssleep8030046 (PMC13510326; doi:10.3390/clockssleep8030046)
Supplement: Supplementary file 1 [file clockssleep-08-00046-s001.zip › clockssleep-4442961-supplementary.pdf]

Table S1. Pictorial-based memory task (number of correct recalls) in the severe OSA subgroup: Improvers (AHI≥30→<30 at 6 months) vs Non-improvers (AHI≥30→≥30). Values are mean (SD). P-values are based on the following statistical tests: (1) independent *t*-test; (2) paired *t*-test; (3) Mann–Whitney test; (4) Wilcoxon signed-rank test. All tests are two-sided ($\alpha = 0.05$); bold indicates $p < 0.05$.

Trial	Improvers (N=95) Baseline mean (SD)	Improvers 6m mean (SD)	Improvers Δ mean (SD)	Within *p* (Improvers)	Non-improvers (N=31) Baseline mean (SD)	Non-improvers 6m mean (SD)	Non-improvers Δ mean (SD)	Within *p* (Non-improvers)	Between *p* (Δ)
**Trial 1**	14,51 (4,16)	16,01 (4,49)	1,51 (4,50)	**<0.001[4]**	12,42 (4,62)	15,29 (5,28)	2,87 (3,33)	**<0,001[2]**	0,185[3]
**Trial 2**	17,59 (4,63)	19,02 (4,50)	1,43 (4,23)	**<0.001[4]**	14,58 (5,61)	17,35 (6,13)	2,77 (3,78)	**<0.001[2]**	0,282[3]
**Trial 3**	19,65 (4,59)	20,05 (4,35)	0,40 (3,67)	0,290[2]	16,97 (5,95)	18,65 (5,61)	1,68 (3,78)	0,071[4]	0,106[1]
**Trial 4**	21,03 (4,55)	21,80 (4,58)	0,77 (4,11)	0,071[2]	19,06 (6,56)	19,42 (5,93)	0,35 (3,06)	0,523[2]	0,552[1]
**Trial 5**	21,14 (4,65)	22,06 (4,79)	0,93 (3,77)	**0,018[2]**	18,52 (7,09)	19,55 (6,59)	1,03 (3,86)	0,146[2]	0,894[1]
**Trial 6**	19,95 (4,75)	21,12 (4,95)	1,17 (3,87)	**0,004[2]**	17,42 (6,39)	19,74 (6,98)	2,32 (3,98)	**0,001[4]**	0,164 [1]

Table S2. Associations between demographic and clinical variables and cognitive improvement over 6 months (Model I). Linear regression coefficients (β)represent the association between each predictor and the change in number of correct recalls (Δ = 6-month – Baseline) in each cognitive trial (1–6). Unadjusted (univariate)models examine each predictor independently. Adjusted (multivariate) models include all predictors simultaneously: treatment status (Treated vs Not-Treated), baseline score, age, and sex. Positive β values indicate greater cognitive improvement. All tests are two-sided ($\alpha = 0.05$). Bold indicates $p < 0.05$.

Severity/Trial / Variable	Multi β	CI95% Inf	CI95% Sup	*p*
**No-OSA /Mild Trial 1**				
**Treatment (T vs NT)**	-1.0700	-2.7410	0.6010	0.213
**Baseline (correct recalls)**	-0.4217	-0.6289	-0.2144	**<0.001**
**Sex (Female *vs.* Male)**	-0.1135	-1.5232	1.7503	0.892
**Age (years)**	-0.0567	-0.1316	0.0181	0.142
**Trial 2**				
**Treatment (T vs NT)**	-0.1909	-1.9448	1.5629	0.831
**Baseline (correct recalls)**	-0.3470	-0.5449	-0.1491	**0.001**
**Sex (Female *vs.* Male)**	1.8694	0.3063	3.4325	0.022
**Age (years)**	-0.0734	-0.1488	0.0020	0.060
**Trial 3**				
**Treatment (T vs NT)**	-1.4625	-2.9582	0.0333	0.059
**Baseline (correct recalls)**	-0.3800	-0.5397	-0.2202	**<0.001**
**Sex (Female *vs.* Male)**	1.3324	-0.0788	2.7436	0.068
**Age (years)**	-0.0494	-0.1176	0.0189	0.161
**Trial 4**				
**Treatment (T vs NT)**	-1.3949	-2.9958	0.2060	0.092
**Baseline (correct recalls)**	-0.4741	-0.6411	-0.3070	**<0.001**
**Sex (Female *vs.* Male)**	0.6604	-0.8733	2.1941	0.401
**Age (years)**	-0.0688	-0.1420	0.0045	0.070
**Trial 5**				
**Treatment (T vs NT)**	-0.2499	-1.7051	1.2054	0.737
**Baseline (correct recalls)**	-0.3648	-0.5044	-0.2252	**<0.001**
**Sex (Female *vs.* Male)**	1.0184	-0.3513	2.3881	0.149

Severity/Trial / Variable	Multi β	CI95% Inf	CI95% Sup	*p*
**Age (years)**	-0.0622	-0.1271	0.0027	0.064
**Trial 6**				
**Treatment (T vs NT)**	-0.8206	-2.2662	0.6249	0.270
**Baseline (correct recalls)**	-0.2882	-0.4230	-0.1533	**<0.001**
**Sex (Female *vs.* Male)**	1.3672	-0.0129	2.7473	0.056
**Age (years)**	-0.0343	-0.0983	0.0296	0.296
**Moderate Trial 1**				
**Treatment (T vs NT)**	0.7369	-0.8231	2.2969	0.359
**Baseline (correct recalls)**	-0.5313	-0.7435	-0.3191	**<0.001**
**Sex (Female *vs.* Male)**	-0.0122	-1.7617	1.7372	0.989
**Age (years)**	-0.0712	-0.1424	0.0000	0.055
**Trial 2**				
**Treatment (T *vs.* NT)**	0.0019	-1.8222	1.8259	0.998
**Baseline (correct recalls)**	-0.6339	-0.8417	-0.4262	**<0.001**
**Sex (Female *vs.* Male)**	1.4267	-0.6126	3.4660	0.176
**Age (years)**	-0.1188	-0.2079	-0.0298	**0.012**
**Trial 3**				
**Treatment (T *vs.* NT)**	0.4194	-1.6029	2.4416	0.686
**Baseline (correct recalls)**	-0.6016	-0.8337	-0.3696	**<0.001**
**Sex (Female *vs.* Male)**	0.8360	-1.4319	3.1038	0.473
**Age (years)**	-0.1080	-0.2023	-0.0136	**0.029**
**Trial 4**				
**Treatment (T *vs.* NT)**	-0.4953	-2.2021	1.2115	0.572
**Baseline (correct recalls)**	-0.5464	-0.7424	-0.3503	**<0.001**
**Sex (Female *vs.* Male)**	1.2926	-0.6200	3.2052	0.191
**Age (years)**	-0.1285	-0.2091	-0.0479	**0.003**
**Trial 5**				
**Treatment (T *vs.* NT)**	-0.7990	-2.1377	0.5398	0.247
**Baseline (correct recalls)**	-0.4250	-0.5663	-0.2838	**<0.001**

Severity/Trial / Variable	Multi β	CI95% Inf	CI95% Sup	*p*
**Sex (Female *vs.* Male)**	0.5564	-0.9376	2.0504	0.469
**Age (years)**	-0.1344	-0.1954	-0.0734	**<0.001**
**Trial 6**				
**Treatment (T *vs.* NT)**	0.1501	-1.5117	1.8118	0.860
**Baseline (correct recalls)**	-0.3157	-0.4910	-0.1404	**<0.001**
**Sex (Female *vs.* Male)**	0.3027	-1.5566	2.1621	0.751
**Age (years)**	-0.1017	-0.1785	-0.0249	**0.012**
**Severe Trial 1**				
**Treatment (T *vs.* NT)**	-1.3611	-3.6037	0.8816	0.237
**Baseline (correct recalls)**	-0.4519	-0.6138	-0.2900	**<0.001**
**Sex (Female *vs.* Male)**	1.3015	-0.4015	3.0046	0.137
**Age (years)**	-0.0788	-0.1436	-0.0140	**0.019**
**Trial 2**				
**Treatment (T *vs.* NT)**	0.1052	-2.0875	2.2978	0.925
**Baseline (correct recalls)**	-0.4011	-0.5476	-0.2546	**<0.001**
**Sex (Female *vs.* Male)**	-0.0395	-1.7476	1.6685	0.964
**Age (years)**	-0.0641	-0.1315	0.0034	0.065
**Trial 3**				
**Treatment (T *vs.* NT)**	-0.7769	-2.7192	1.1655	0.435
**Baseline (correct recalls)**	-0.3655	-0.4889	-0.2421	**<0.001**
**Sex (Female *vs.* Male)**	0.2788	-1.2067	1.7643	0.714
**Age (years)**	-0.0480	-0.1053	0.0093	0.104
**Trial 4**				
**Treatment (T *vs.* NT)**	-0.1577	-2.2368	1.9214	0.882
**Baseline (correct recalls)**	-0.3397	-0.4697	-0.2098	**<0.001**
**Sex (Female *vs.* Male)**	0.6235	-0.9640	2.2111	0.443
**Age (years)**	-0.0416	-0.1034	0.0203	0.191
**Trial 5**				
**Treatment (T *vs.* NT)**	0.0123	-2.0396	2.0641	0.991

Severity/Trial / Variable	Multi β	CI95% Inf	CI95% Sup	*p*
**Baseline (correct recalls)**	-0.2798	-0.4023	-0.1574	**<0.001**
**Sex (Female *vs.* Male)**	-0.3712	-1.9559	1.2135	0.647
**Age (years)**	-0.0529	-0.1140	0.0083	0.093
**Trial 6**				
**Treatment (T *vs.* NT)**	-0.7964	-2.9480	1.3552	0.470
**Baseline (correct recalls)**	-0.2264	-0.3560	-0.0968	**<0.001**
**Sex (Female *vs.* Male)**	-1.3533	-3.0151	0.3085	0.113
**Age (years)**	-0.0506	-0.1129	0.0118	0.115

Table S3. Associations between physiological sleep variables and memory task improvement over 6 months. Linear regression coefficients (β) represent the association between each physiological or polysomnographic variable and the change in number of correct recalls (Δ = 6-month – Baseline) in each cognitive trial (1–6). Each variable was tested in a separate multiple linear regression model adjusted for treatment status (Treated vs Not-Treated), baseline score, sex, age, and BMI. Positive β values indicate greater cognitive improvement. All tests are two-sided (α = 0.05); bold indicates p < 0.05.

SEVERITY/TRIAL/VARIABLE	MULTI B	CI95% INF	CI95% SUP	*p*
**NO-OSA/MILD · TRIAL 1**				
**Treatment (T *vs.* NT)**	-1.6577	-3.8844	0.569	0.153
**Baseline (correct recalls)**	-0.3276	-0.5552	-0.1	**0.008**
**Sex (female *vs.* male)**	0.2754	-1.5986	2.1494	0.775
**Age (years)**	0.0145	-0.0723	0.1013	0.745
**BMI (baseline)**	-0.0685	-0.3071	0.1701	0.577
**NREM sleep (% TST, baseline)**	0.1496	0.0043	0.2949	0.051
**Stage N2 (% TST, baseline)**	0.0916	-0.0181	0.2014	0.111
**Respiratory Arousal Index (baseline)**	-0.2339	-0.5444	0.0767	0.149
**Spontaneous Arousal Index (baseline)**	-0.2124	-0.3829	-0.0419	**0.020**
**NO-OSA/MILD · TRIAL 2**				
**Treatment (T *vs.* NT)**	-0.0947	-1.8711	1.6817	0.917
**Baseline (correct recalls)**	-0.3494	-0.548	-0.1508	**<0.001**
**Sex (female *vs.* male)**	1.8163	0.2428	3.3899	**0.027**
**Age (years)**	-0.0723	-0.1479	0.0034	0.065
**BMI (baseline)**	-0,0658	-0,2360	0,1044	0,451
**NO-OSA/MILD · TRIAL 3**				
**Treatment (T *vs.* NT)**	-1,2095	-2,5405	0,1215	0,084
**Baseline (correct recalls)**	-0,3939	-0,5199	-0,2679	**<0,001**
**Sex (female *vs.* male)**	2,3349	1,1017	3,5680	**<0.001**
**Age (years)**	-0,0245	-0,0786	0,0297	0,383
**BMI (baseline)**	-0,0117	-0,1247	0,1012	0,840
**Time SpO2<85% (% time, baseline)**	-1,7045	-2,4297	-0,9793	**<0.001**
**NO-OSA/MILD · TRIAL 4**				

SEVERITY/TRIAL/VARIABLE	MULTI B	CI95% INF	CI95% SUP	p
Treatment (T *vs.* NT)	-0,4455	-2,2755	1,3846	0,637
Baseline (correct recalls)	-0,4069	-0,5741	-0,2396	**<0,001**
Sex (female *vs.* male)	0,2528	-1,5207	2,0263	0,782
Age (years)	-0,0298	-0,1062	0,0465	0,449
BMI (baseline)	-0,0281	-0,1889	0,1327	0,734
WASO (min, baseline)	0,0232	0,0013	0,0452	**0,046**
Time SpO2<85% (% time, baseline)	-1,8844	-2,9649	-0,8038	**0.002**
**NO-OSA/MILD · TRIAL 5**				
Treatment (T *vs.* NT)	-0,0155	-1,4746	1,4435	0,983
Baseline (correct recalls)	-0,3733	-0,5111	-0,2355	**<0,001**
Sex (female *vs.* male)	0,9377	-0,4263	2,3017	0,182
Age (years)	-0,0467	-0,1119	0,0185	0,165
BMI (baseline)	0,0118	-0,1419	0,1654	0,881
Time SpO2<90% (min, baseline)	-0,0154	-0,0291	-0,0018	**0,030**
**NO-OSA/MILD · TRIAL 6**				
Treatment (T *vs.* NT)	-1,2474	-3,7913	1,2965	0,349
Baseline (correct recalls)	-0,3312	-0,6078	-0,0546	**0,031**
Sex (female *vs.* male)	1,3170	-1,7582	4,3922	0,412
Age (years)	-0,0473	-0,1890	0,0943	0,521
BMI (baseline)	-0,1411	-0,4395	0,1572	0,366
Obstructive Apnea Mean Duration (s, baseline)	0,3889	0,0729	0,7049	**0,027**
**MODERATE· TRIAL 1**				
Treatment (T *vs.* NT)	0,6184	-0,9114	2,1482	0,432
Baseline (correct recalls)	-0,5373	-0,7447	-0,3298	**<0,001**
Sex (female *vs.* Male)	0,4828	-1,3035	2,2691	0,599
Age (years)	-0,0593	-0,1300	0,0113	0,106
BMI (baseline)	-0,1685	-0,3444	0,0074	0,066
**MODERATE· TRIAL 2**				

SEVERITY/TRIAL/VARIABLE	MULTI B	CI95% INF	CI95% SUP	*p*
**Treatment (T *vs.* NT)**	0,2047	-1,3589	1,7682	0,799
**Baseline (correct recalls)**	-0,6631	-0,8458	-0,4804	**<0,001**
**Sex (female *vs.* Male)**	2,0705	-0,4186	4,5596	0,114
**Age (years)**	-0,1295	-0,2119	-0,0471	**<0,005**
**BMI (baseline)**	-0,1389	-0,3541	0,0764	0,217
**Stage N1 (% Tst, baseline)**	-0,1708	-0,4001	0,0585	0,155
**Stage N2 (min, baseline)**	0,0215	0,0025	0,0405	**0,035**
**Spontaneous Arousal Index (baseline)**	-0,1859	-0,3007	-0,0710	**0,0043**
**SpO2 Minimum (baseline)**	-0,1808	-0,3732	0,0116	0,076
**MODERATE· TRIAL 3**				
**Treatment (T *vs.* NT)**	0,2713	-1,5792	2,1219	0,775
**Baseline (correct recalls)**	-0,5412	-0,7557	-0,3268	**<0,001**
**Sex (female *vs.* male)**	0,8200	-1,3712	3,0113	0,467
**Age (years)**	-0,0450	-0,1377	0,0476	0,345
**BMI (baseline)**	-0,1486	-0,3631	0,0659	0,180
**Time SpO2<90% (% time, baseline)**	-0,0907	-0,1475	-0,0339	**0,003**
**MODERATE· TRIAL 4**				
**Treatment (T *vs.* NT)**	-0,0519	-1,4614	1,3575	0,943
**Baseline (correct recalls)**	-0,4201	-0,5923	-0,2480	**<0,001**
**Sex (female *vs.* Male)**	0,3226	-1,4084	2,0536	0,717
**Age (years)**	-0,0907	-0,1596	-0,0218	**0.013**
**BMI (baseline)**	-0,0207	-0,1895	0,1480	0,811
**Latency to rem (min, baseline)**	-0,0175	-0,0301	-0,0049	**0,009**
**SpO2 minimum (baseline)**	-0,3607	-0,5362	-0,1853	**<0.001**
**Time SpO2<90% (min, baseline)**	-0,0275	-0,0399	-0,0151	**<0.001**
**MODERATE· TRIAL 5**				
**Treatment (T *vs.* NT)**	-0,2743	-1,7377	1,1891	0,715
**Baseline (correct recalls)**	-0,5594	-0,7279	-0,3910	**<0.001**
**Sex (female *vs.*male)**	2,2511	0,1290	4,3732	**0,045**

SEVERITY/TRIAL/VARIABLE	MULTI B	CI95% INF	CI95% SUP	*p*
Age (years)	-0,1406	-0,2083	-0,0729	**<0.001**
BMI (baseline)	-0,3155	-0,5014	-0,1296	**0,002**
Respiratory Arousal Index (baseline)	-0,2143	-0,3606	-0,0679	**0,007**
**MODERATE· TRIAL 6**				
Treatment (T *vs.* NT)	0,3626	-1,2314	1,9565	0,658
Baseline (correct recalls)	-0,3350	-0,5026	-0,1675	**<0.001**
Sex (female *vs.* Male)	-0,4077	-2,3295	1,5142	0,679
Age (years)	-0,0875	-0,1623	-0,0128	**0,026**
BMI (baseline)	-0,0208	-0,2032	0,1616	0,824
Stage N1 (% TST, baseline)	-0,3049	-0,5217	-0,0881	**0,008**
**SEVERE· TRIAL 1**				
Treatment (T *vs.* NT)	-1,0717	-3,3239	1,1805	0,353
Baseline (correct recalls)	-0,4604	-0,6215	-0,2993	**<0.001**
Sex (female *vs.* male)	1,5868	-0,1368	3,3104	0,074
Age (years)	-0,0809	-0,1452	-0,0165	**0,015**
BMI (baseline)	-0,1146	-0,2493	0,0201	0,098
**SEVERE· TRIAL 2**				
Treatment (T *vs.* NT)	0,6361	-1,5821	2,8543	0,575
Baseline (correct recalls)	-0,3442	-0,4941	-0,1942	**<0.001**
Sex (female *vs.* male)	0,1555	-1,5488	1,8598	0,858
Age (years)	-0,0465	-0,1134	0,0203	0,175
BMI (baseline)	-0,0569	-0,1901	0,0762	0,404
Hypopnea Mean Duration (s, baseline)	-0,1271	-0,2318	-0,0224	**0,019**
Mixed Apnea Index (baseline)	0,0562	-0,0001	0,1125	0,053
**SEVERE· TRIAL 3**				
Treatment (T *vs.* NT)	-1,3922	-5,1542	2,3698	0,473
Baseline (correct recalls)	-0,3686	-0,5755	-0,1617	**0,001**
Sex (female *vs.* male)	1,0349	-3,9231	5,9928	0,685
Age (years)	-0,0533	-0,1447	0,0381	0,260

SEVERITY/TRIAL/VARIABLE	MULTI B	CI95% INF	CI95% SUP	*p*
**BMI (baseline)**	-0,0221	-0,2979	0,2537	0,876
**Mixed Apnea Longest Duration (s, baseline)**	-0,0624	-0,1290	0,0041	0,073
**SEVERE· TRIAL 4**				
**Treatment (T *VS.* NT)**	-1,4199	-4,0309	1,1911	0,291
**Baseline (correct recalls)**	-0,2892	-0,4362	-0,1423	**<0.001**
**Sex (female *vs.* male)**	-0,3997	-2,7996	2,0003	0,745
**Age (years)**	-0,0694	-0,1323	-0,0065	**0,035**
**BMI (baseline)**	-0,1018	-0,2839	0,0803	0,278
**Obstructive Apnea Mean Duration (s, baseline)**	-0,1554	-0,2969	-0,0140	**0,036**
**SEVERE· TRIAL 5**				
**Treatment (T *VS.* NT)**	0,6301	-1,4178	2,6780	0,548
**Baseline (correct recalls)**	-0,2560	-0,3768	-0,1352	**<0.001**
**Sex (female *vs.* male)**	-0,1699	-1,7243	1,3845	0,831
**Age (years)**	-0,0393	-0,0988	0,0202	0,198
**BMI (baseline)**	-0,0829	-0,2063	0,0406	0,191
**Latency to REM (min, baseline)**	-0,0110	-0,0195	-0,0025	**0,013**
**Hypopnea Mean Duration (s, baseline)**	-0,1230	-0,2172	-0,0287	**0,012**
**SEVERE· TRIAL 6**				
**Treatment (T *VS.* NT)**	-0,7848	-2,9708	1,4011	0,483
**Baseline (correct recalls)**	-0,2267	-0,3573	-0,0962	**<0.001**
**Sex (female *vs.* male)**	-1,3414	-3,0445	0,3616	0,125
**Age (years)**	-0,0506	-0,1134	0,0121	0,116
**BMI (baseline)**	-0,0046	-0,1357	0,1266	0,946
